# An Interview with Dennis Hong

**DOI:** 10.1089/rorep.2023.29006.intdh

**Published:** 2023-12-11

**Authors:** Marwa ElDiwiny

**Affiliations:** Marwa ElDiwiny, Vrije Universiteit Brussel, Brussels, Belgium. Dennis Hong, Mechanical & Aerospace Engineering Department, University of California, Los Angeles, Los Angeles, California, USA.

**Figure:**
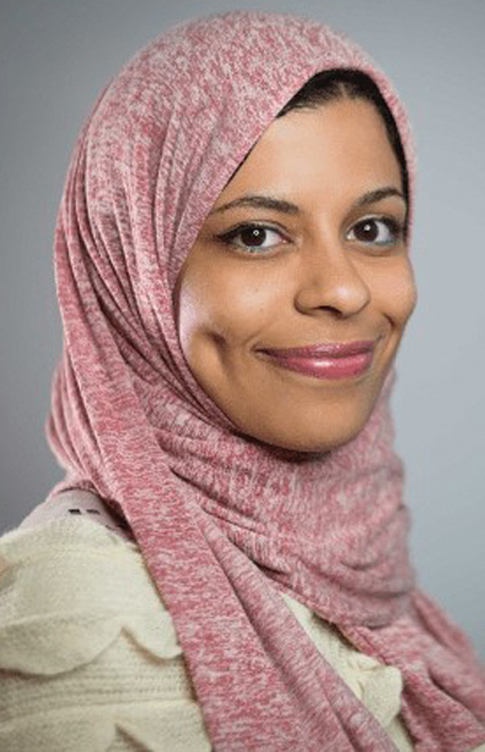
Marwa ElDiwiny

**Figure:**
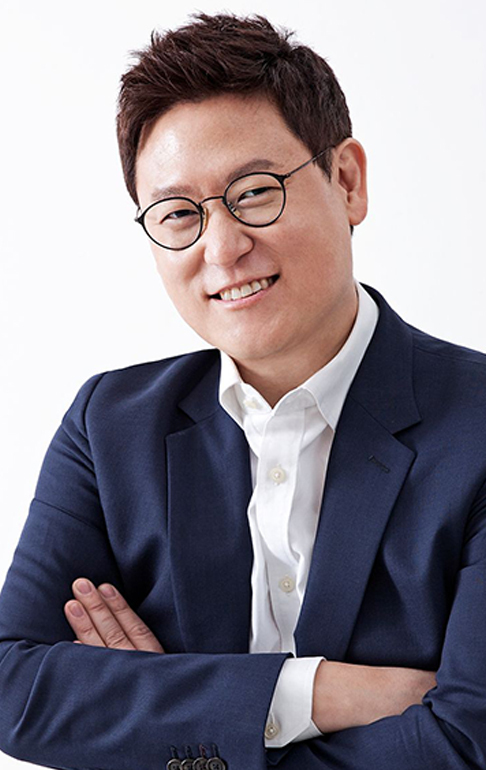
Dennis Hong

***Marwa ElDiwiny:* Please describe the design of the feet on your humanoid robots.**

**Dennis Hong:** Our previous humanoid robots had big, flat plates as feet, and those are good if you're doing position control on smooth, hard, even surfaces, but if it's not that kind of condition, then they're not good. As you can imagine, with a rough surface and a rigid plate, your contact point is unpredictable and it changes. With the more recent generation of robots, dynamic robots, you see a lot of pointed feet, and because it's a point contact, it's much easier to model.

We wanted to go a little bit beyond that, so we have small feet, the size of a finger or a pen. That was our original design, and we have a silicone molding case, like a slipper over that finger-sized foot. The feet are very slippery, so one day we said, “Athletic shoes, sneakers are designed for these kind of tasks.” They're optimized for that, so why not just use sneakers? We put on a sneaker. We just needed to 3D print out a simple insert from that aluminum, metal, finger-sized foot to the foot shape. We put a shoe on, it walks, and it walks much better. The grip is great, much more stable, much faster.

If you've been in this field of bipedal locomotion, you probably know that every time you change the slightest thing, link length, mass properties, something different, you have to change all the parameters and you have to modify everything. It takes hours, if not days to optimize everything.

But ARTEMIS was different. The change from a finger-size foot to a sneaker, it's a huge difference, but we just put it on and it worked. Now Nike sends us shoes for free. If you've seen our latest videos of ARTEMIS, it's always wearing Nikes. Currently, it's wearing the very popular Dunk Lows, we call it Pandas, the black-and-white Nike shoes. That's her favorite right now.

Before I'm a roboticist, I'm a loving father and a husband. That's more important, and of course I'm a roboticist. Doing research is important, but I would like to be remembered more as an educator. In our laboratory, RoMeLa, the Robotics Mechanism Laboratory at UCLA, we build robots, we do research, we publish articles, but the most important outcome, the most important things from our laboratory is not our articles, it's not our robots, it's our students. I want to make the world a better place by pushing out great students who are going to be leaders in the field in robotics and engineering.

**Figure:**
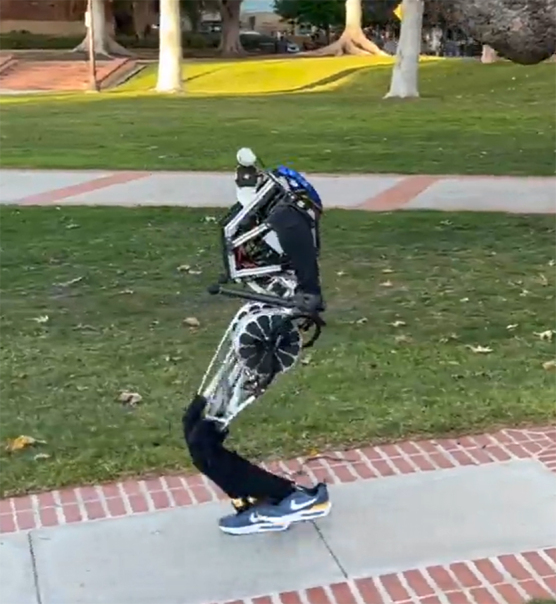
ARTEMIS

***Marwa ElDiwiny:* Before we talk more about ARTEMIS, I wanted to ask you about what is missing in robotics. You have been in the field I think a long time, and you see a lot of iteration. What's still missing in robotics?**

**Dennis Hong:** This might sound weird, but really everything. Robotics is such an integrated field that you need everything, and every time you have a breakthrough, you start to see that things are missing. For example, we have built a lot of hardware prototypes that move, and they only last for 30 minutes. Of course, we need to have better energy sources, and people are working on that.

We also do a lot of leg locomotion, walking on two legs, three legs, four legs, or six legs, and because of that, we need better actuators. Most of our robots that we use today use electric-based actuators, are server controls that do position control. We need better torque force-control compliant actuators like proprioceptive actuators. We have new technology, but we need different types of more biologically inspired actuators. We need better materials, lighter weight, more strength-to-weight ratio. More computing power is always great, so again, we need everything.

***Marwa ElDiwiny:* The most interesting part I think in ARTEMIS is the actuation, and the artificial muscle resembling the biological artificial muscle. Can you tell us some more about the design?**

**Dennis Hong:** Most of the robots that exist today are stiff, position-controlled robots, the industrial robots. These are great for precise motion such as assembly tasks. However, when we want to have robots living and working next to humans, we need to have something safer, and thus we need to have compliant joints, and especially for applications where it goes through a lot of impact or contact, then we need these kinds of biological characteristic actuators.

For the general public, I often use the term artificial muscles, but that could be a little bit misleading. These are not really biology based or chemical based. Basically, these are proprioceptive actuators. Professor Sangbae Kim from MIT started this revolution with the MIT cheetah robot. Regular position servos are electric motors with a huge gear ratio gear output. Motors are really fast, but very weak in torque. What we need is to slow it down and increase the torque, thus we have these huge gear boxes or harmonic drives that like a 200 to 1, 300 to 1 gear ratio that will up the torque and lower the speed, and that's great for position control.

Proprioceptive actuators are a little bit different. We now have these higher torque motors that we call pancake motors, that have really high diameters. The best scenario is not to have any gears at all, but of course in reality we need to have some kind of gear. We have a one to four, one to six gear ratio, really low gear–ratio gearbox, and that's the output, so those are basically proprioceptive actuators.

The advantage is because the gear ratio is so small, it's back drivable, which means that conventional servo motors, if you turn off the power, it doesn't move, it's stiff. You cannot do a torque control, a sense of torque from the motor side. If you have a very low gear ratio, then when you turn off the power, for example, then it's going to flop down because it's back drivable. If that's the case, we can use the current from the motor to do direct torque control and that's the benefit. If you use these kinds of actuators, you can easily do torque control, it's great for impact mitigation. You can do so many things. You can change all the physical parameters just by software, and I think that's the greatest advantage.

There are some other approaches as well. Hydraulics is one way to do something similar to that. Another way is what we call that a series elastic actuator, which is basically a push-pull servo and at the end we have a compliant spring or some kind of rubber or some kind of compliant element to it. Previously we used many series elastic actuators. It solves a problem, but it creates another one. For example, the control bandwidth is bad, pretty low. Proprioceptive actuator is not the best solution. We need better ones, but at this point I believe that's the best way to go with leg robots that go through impact.

***Marwa ElDiwiny:* In your TED Talks, you highlight the evolution of the robots you design. It's cool that you go for BALLU design and then you go to humanoid robot. When you have a problem, you shift to a new idea. Can you tell us about the iteration and how it's evolved in your laboratory?**

**Dennis Hong:** I have a dream. In the future, I would like to be living with robots. Robots doing the dishes, taking out the trash. It's all great, but I claim that for robots to be living in our environment, the robot needs to be human shape and human size, and that's why we need humanoid robots, because if you look around, your stairs are a certain height for robots to walk up, your door handle is a certain height for humans to open up, or for robots to use tools, the robots need to be human shape and size, and that's why we've been working on humanoid robots for a long time. For the past 20 years, I've been working with humanoid robots.

**Figure:**
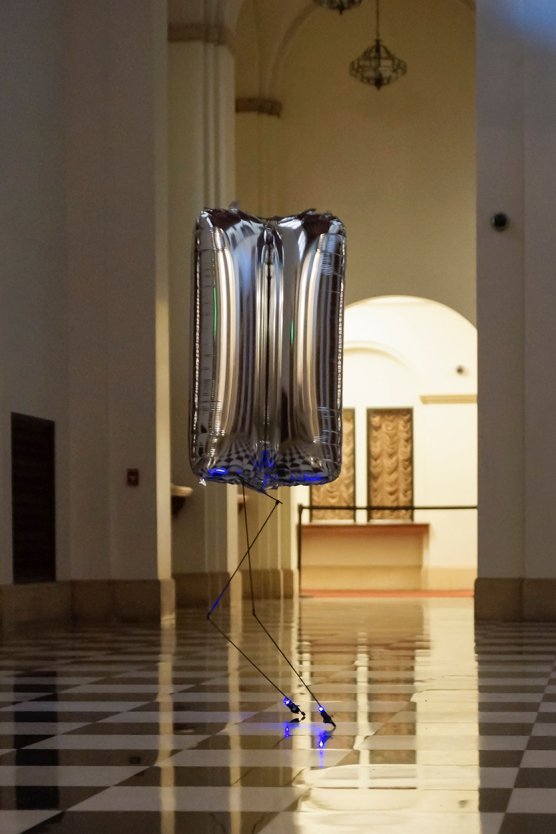
BALL-U

**Figure:**
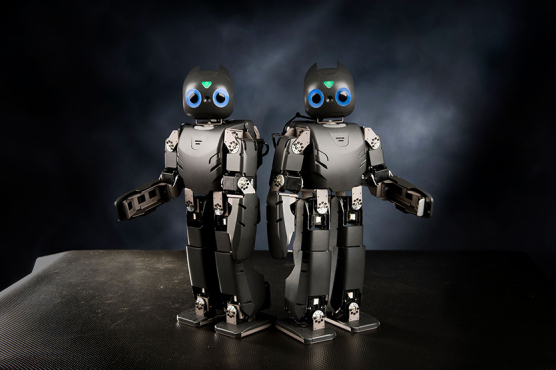
DARwin-OP

**Figure:**
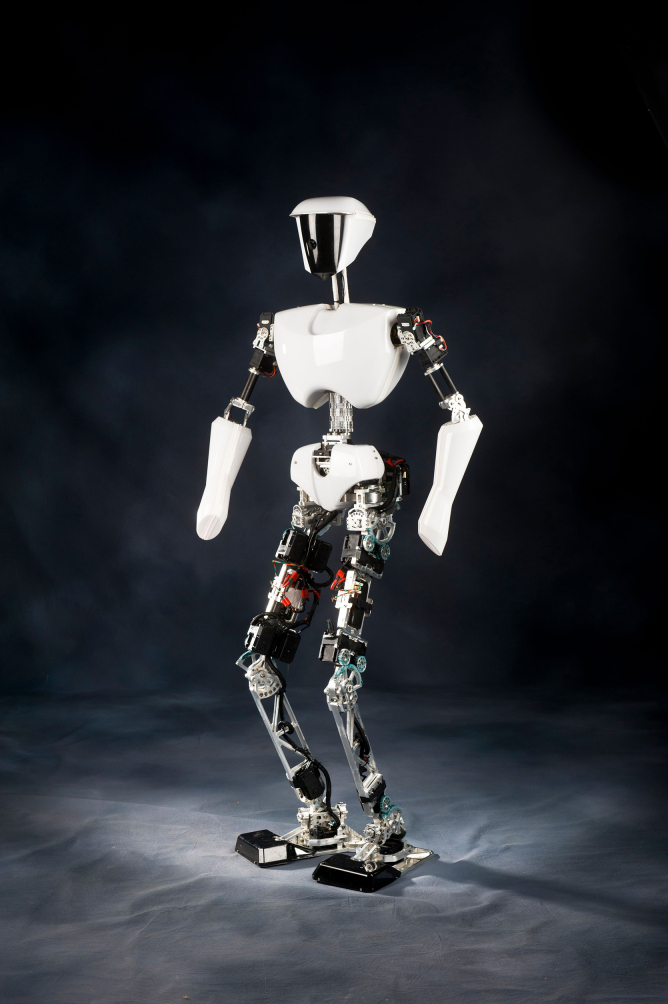
CHARLI

One of the early humanoid robots that we designed is called DARwin-OP. It's an open-source research education robot. CHARLI is considered for the United States the very first humanoid robot. THOR-RD was used for the DARPA robotics challenge. SAFFiR is a firefighting humanoid robot on navy ships. We also developed THOR, and many, many different types of big and small robots, probably more than a dozen different types of robots that we developed in our laboratory.

These have had many problems. We all know that they constantly fall down, they're slow. We now have technology that we can use to solve these kinds of problems, but they're still very, very expensive. They're very complex and I'm sure that you agree, they're very dangerous. You don't want to be next to these robots, big robots moving around. Is there a way to make these biped robots solve all these problems?

Consider your typical humanoid robot. When the robot walks forward, one of the reasons why it constantly falls out is because the left and right legs have this distance, and because the legs go up and down and forward and backward. Because of this, it creates this unwanted torque or moment, that's what causes it to fall down. The RoboOne Competition is especially popular in Japan. It's a robot fighting competition. If you look at these robots, they are very fast and agile and they don't fall down. If you look carefully, they walk sideways, because if you walk sideways, what happens? Your left and right legs, they line up. The twisting moment disappears. I got this idea from watching TV, fencing, or ballet, they always walk sideways.

**Figure:**
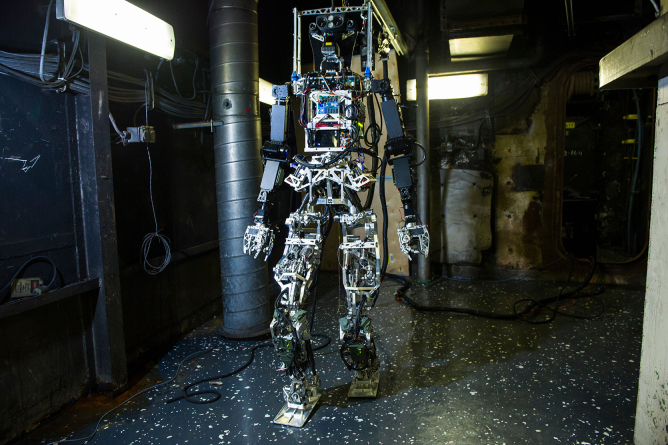
SAFFiR

**Figure:**
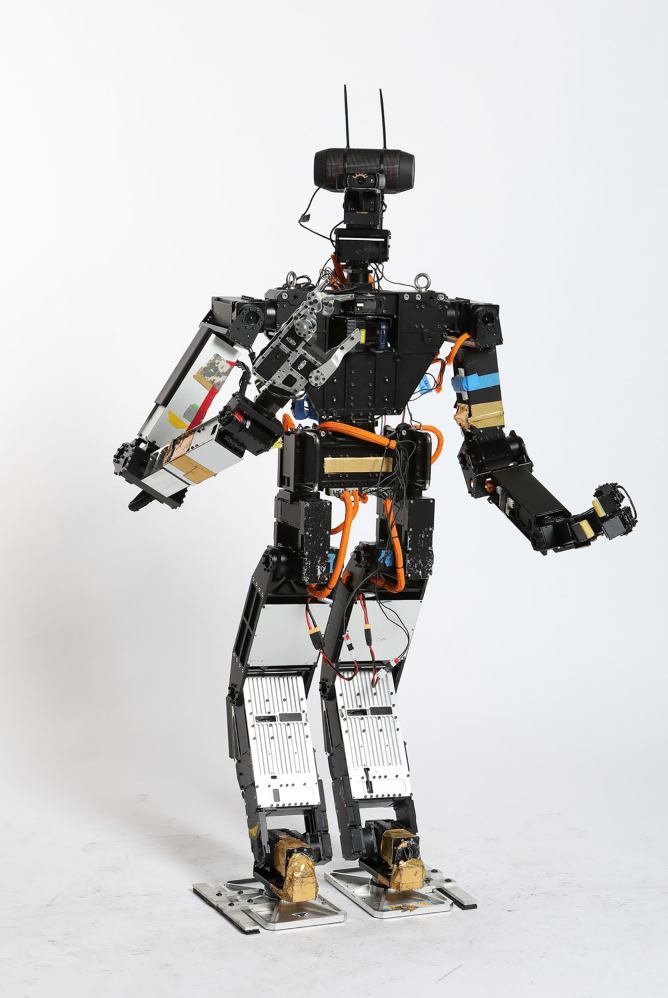
THOR-RD

We evolved the walking motion when we developed NABi, Non-Anthromorphic Biped. Simply changing the orientation of the legs, solved all the problems. It doesn't fall down, it's ridiculously simple, it's ridiculously stable. It can also jump up. We solved a lot of problems, but the problem is this can walk forward and backward, but it cannot change directions. So, what do we do?

Then, let's have two of these NABi stacked onto each other, and that is called ALPHRED, Autonomous Legged Personal Help Robot with Enhanced Dynamics. It can walk with two legs like NABi, it can walk with four legs, it can change its configuration, it can do many things. So, then we developed this actuator called the BEAR actuator that we just talked about, Back-drivable Electromagnetic Actuator Robots. We replace the actuators for the NABi with these and now we have NABi-2. This one can now not only walk, but also can hop and jump. It's great, it can do all these things. We're trying to do jump rope. Now it's time to change ALPHRED with this BEAR actuator, so we have ALPHRED2.

**Figure:**
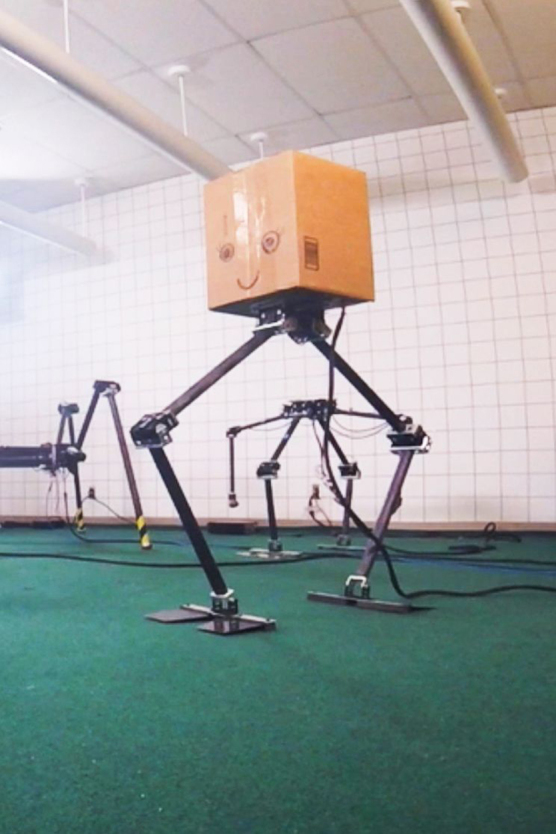
NABi

Now it's really agile, it walks very fast and stable. It can hop and jump, it can walk with two feet. It can use its other two limbs for picking up objects. This shows our evolution of ideas. We have an idea and then it's causing problems. How do we solve this? We come up with constantly creative ideas, but again, you can also use it for a useful task.

**Figure:**
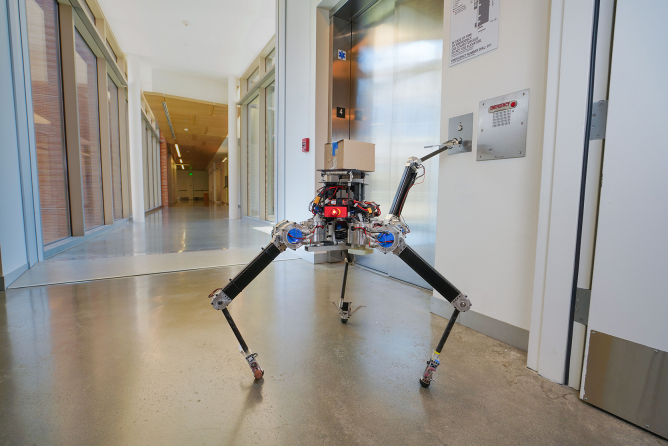
ALPHRED-2

***Marwa ElDiwiny:* What did you learn from all these? Before going to ARTEMIS, what is the common thing that you learned?**

**Dennis Hong:** What I learned is this, there are always hurdles. When you're doing research, if you know that there's a solution, we don't call that research. If you know there's a solution, we call that homework. Research is something that you do not know if it's possible or not, but you go for it. That is what we call research. In our laboratory, we do not do our homework, we do our research. We do not know if it's going to work, but we give it a try, and so many times I teach this to our students. This is where I am, and I know my destination. I want to go there, I'm going hiking.

Sometimes there's a big puddle of water or there's a cliff that I need to go around. There's a huge mound that it's difficult to climb, so I go around, but you go around and while doing that, even though I know my destination is over there, sometimes I find something new, something different, or sometimes I find a completely different place that is better than the destination that I wanted to go to.

Then, that could be a better solution. We are not afraid to challenge different things and try new things. If there's a failure or things don't work out, that's fine. We take that, we learn from it because if you have a failure, if you give up, then that's the end of the story, but if you use that as the learning experience, it becomes a steppingstone for success, and that's what we do in our laboratory, and that's how we come up with innovation.

***Marwa ElDiwiny:* Excellent, now I would like to talk about ARTEMIS.**

**Dennis Hong:** ARTEMIS stands for Advanced Robotic Technology for Enhanced Mobility and Improved Stability. It's a humanoid robot that is our latest humanoid robot that we are very, very excited about. I have to be honest, I've been doing humanoid robot research for more than 20 years, and again, I still truly believe, I always tell people that I would like to have robots living in our home, and to do that, I believe that robots need to be human shape and size. However, if you asked me this question 3 years ago, I would've said, that's still our goal, that's my dream, but there's a chance that this is possibly not going to happen during my lifetime, but we need to start now to make it happen sometime. My expectation of when was very, very conservative.

I thought it's going to happen really far in the future. However, with the success of ARTEMIS and how it's performing, I'm starting to change my thoughts and maybe the humanoid robots living with us are much closer than at least what I've been thinking. ARTEMIS is a humanoid robot. It's a constant evolution for the past 20 years from DARwin, CHARLI, THOR, THOR-RD, SAFFiR, all these robots, but this robot uses that proprioceptive actuator that I just talked about. It's compliant actuation, it can run, it can walk 2.1 m per second.

Correct me if I'm wrong, but I believe this is the fastest walking humanoid robot in the world so far. It might be the fastest running humanoid robot, but we just don't have enough space to test it. Just last week, we started to bring it outdoors in the UCLA campus in the soccer field, the track field, and we're starting to do experiments outdoors, but we believe that this might be the fastest running robot as well.

Boston Dynamics' Atlas robot is the greatest. Everybody agrees that, at this point, it's the greatest robot that exists today, but I make a bold claim that our humanoid robot could be a contender for Atlas. The Atlas robot uses hydraulics, so there's no way ARTEMIS can be stronger, more powerful. However, in terms of speed or usability, maybe. But this robot can walk jump, it moves boxes, it can push, kick. They can walk on a rubble pile. We take it outdoors on campus, it walks on the grass without tethers by the way—and that’s a big thing. Doing robot experiments outdoors without tethers—that’s a statement—so we’re at that point right now.

***Marwa ElDiwiny:* Excellent. What are some of the attributes of the ARTEMIS?**

**Dennis Hong:** ARTEMIS is very robust. It is the five-time world champion in the RoboCup competition, the international robot soccer competition. It's autonomous. It walks up to 2.1 m per second. It can also run. As you know, running means that there's a flight phase, with two feet off the ground. Running is not yet that stable, so we still have a lot of work to do, but it does run without tethers. It can push things. It doesn't have any hands right now.

**Figure:**
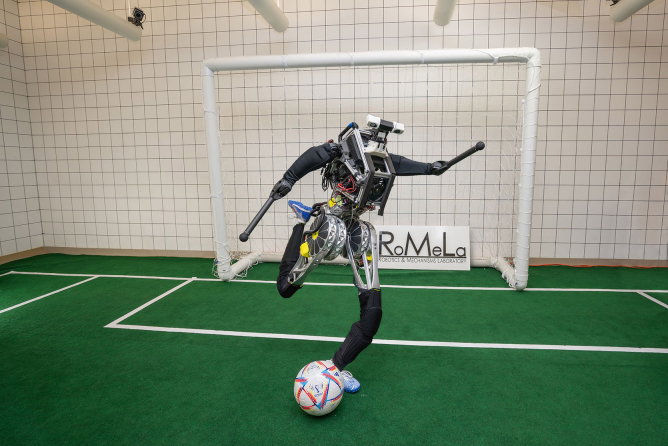
ARTEMIS

***Marwa ElDiwiny:* That's impressive. Can you tell us about the optimization of the leg design?**

**Dennis Hong:** So, let's start with the easy stuff first. Of course, the audience are roboticists, but you probably all know this, but generally for the layperson, the general public, when I talk about bipedal robots, they're constantly falling down on the instability. The layperson might think, “Why don't you lower the center of gravity and make the feet heavy, then it's going to be stable?” Or they might say, “Why did you make the foot so small?.” Bigger feet would make the structure more stable if it were a static structure, but this is a dynamic moving robot. For these types of bipedal robots, you want to have the feet as light as possible.

We tried to reduce the mass and to bring the center of gravity toward the belly button, so to speak. To do that, we used different techniques. For example, typically a humanoid robot leg, a single leg has six degrees of freedom. Most humanoid robots have that, but with ARTEMIS, we have five degrees of freedom. We intentionally gave up on one degree of freedom and that's the roll of the ankle. The foot only has pitch, it doesn't have roll, which I think is okay for what we're trying to do. The reason why we got rid of one degree of freedom is because we wanted to reduce the distal mass.

We're also trying to pull most of the heavy things, which are the actuators, toward the center. If you look at the hip, you see that there are big disks. Those are the big BEAR actuators, two for the left hip and two for the right hip. One is for the leg pitch, the other one is for the knee. We have a linkage system to bring the actuator for the knee up toward the hip. Same thing for the pitch for the ankle; we move it toward the knee. We try to move heavy stuff upstream, so to speak.

Next is something that is a brand new technology, you cannot buy components. Every single part we design and build is in-house. We have a bi-axis CNC machine. We cut our own gears, we designed our power electronics, motor controllers, everything except for bolts and nuts, cameras, and computers, we design everything, including our foot contact access. The reason why these parts, like this femur, look so organic is because we use topology optimization when we design things.

In the computer CAD, we give all the possible boundary volume, and all the loading conditions. It spits out an optimized topology, and based on that we go through iteration, and the result of that the rig step is very organic and it works perfectly. It's the lightest weight for the structure.

***Marwa ElDiwiny:* Can you comment on the noise related to the energy source?**

**Dennis Hong:** One disclaimer, I'm a mechanical engineer, that's my field of study. I've never developed a robot using hydraulics. My experience and knowledge are based on what I've learned and what I've seen with my colleagues working with hydraulic systems. With that disclaimer, hydraulic systems at this point, if you want power, are the way to go because electric-based systems still cannot beat in terms of power density hydraulics, and I believe that that's why Boston Dynamics is using hydraulics. They have their new type of hydraulic borders, which are fantastic, but again, with my understanding of hydraulic systems, it becomes very difficult to design and package.

You need to allow this plumbing, you need to have an accumulator, you need to have the compressor, and this compressor is very, very noisy. Energy efficiency is also a problem, so it has its own share of problems. It's also very messy. It's very difficult to avoid leakage for hydraulic systems. It's not that practical for indoor use or next to humans, but again, if you're looking at outdoors, your crane and these excavators, they use hydraulics. It's good for outdoor big, messy stuff. My goal is to have robots living with us in this human environment. Thus, we want to go with an electric motor-based system, so that's what we're doing. If you've seen some of our ARTEMIS robot videos, many people say, “It makes this… clack, clack, clack, clack, clack, sound.”

Because our robot videos are very fascinating, once you start watching, you cannot take your eyes off of them. In your head, even after you turn off the video, you constantly hear this clack, clack, clack, sound. Many people think that clack, clack sound is from the feet hitting the ground, but that's not the case. The feet hitting the ground, that's fine. It's really the BEAR actuator because it's a rotary actuator, it needs to instantly change the direction, and that's from the backlash from the gear. That's where the sound happens. Because we designed and we manufacture all the gears in-house by ourselves, it cannot be perfect. I think that's where we can make improvements, but again, the sound for me it's like music. We dance to the beat.

***Marwa ElDiwiny:* What are the next steps for ARTEMIS? What's your next plan or improvement?**

**Dennis Hong:** Many things. I think ARTEMIS is the beginning of something great, but still, we have a long way to go. We are pretty much satisfied with the walking locomotion. I think it's stable enough to be used safely in public spaces around people. That's why we bring it out in the campus and things like that, but we want to break the Guinness World Record for the fastest running humanoid robot, and we believe it might already be fast enough to do that. We just haven't tested it for running compared with walking.

But it is going to fall, and when it falls, we want to make it fall safely. Once it has fallen, it needs to stand up on its own, so we're also looking into that. Mechanically, of course, it's designed to do parkour, jumping, and flipping. Mechanically, it has the power, the strength to do all these crazy hyperdynamic locomotion, but again, it's going to take time to utilize the full power of it. We're still using less than 40% of its power, so, we still can do many things. The robot has the mechanical ability to climb up stairs, but we have not implemented that yet. Climbing stairs would be something that we want to work on.

It does have an upper body, the arms and head move, but the arms are really not really being used. We do try to do counterbalance for stability, but it turns out that the arms, the energy, are so low it doesn't have much effect, so that's not useful, but it's not designed for manipulation. I would say our focus is on the locomotion right now. Manipulation is not our current focus, but of course, if you want to have these robots do real useful tasks, it needs to do manipulation. The robot does not have any hands, so probably we're going to put on hands. We do have many hands developed in-house, but we are not going to put hands on ARTEMIS until we believe that it's ready to do meaningful manipulation.

Even with these rubber ball things, it can pick up boxes and move them.

***Marwa ElDiwiny:* How do you imagine robot to be more robust in failure, like falling?**

**Dennis Hong:** There are two things. One is, if it falls, how to make it fall safely, and before that is trying to make it not fall at all. Trying to not fall at all is what we're trying to do, try to make it robust, resistant against external disturbance. We've been working on that, and again, as I mentioned, were happy with where we are. Now we want to focus on how to make it fall safely. If you think about humans, there are many different techniques, such as using your arms like a flywheel.

Sometimes when you slip, you jump or take a big step to rebalance yourself, or sometimes you cannot do anything then you just don't. Most E-stop switches just cut the power, but that's not how it should be. In an emergency, it should have a damping mode, so you fall down slowly or when you fall down, you probably can tuck in your body like this or possibly brace yourself. There are many different techniques, many approaches that we can employ, and those are some of the research tests that we want to do.

***Marwa ElDiwiny:* Excellent. What is your view of the humanoid robot market?**

**Dennis Hong:** It's a little bit of a sensitive topic for me to publicly say things about because I might or might not be involved with a certain company and things like that, but as I mentioned, if you asked me 3 years ago, I would have said, “Humanoid robots don't make any sense as a market right now. They're still dangerous, they're expensive.” Now I'm starting to change my thought a little bit, but still as a product, I'm not sure.

Many smart people, successful business people, are starting to get involved. Boston Dynamics is more like a research long-term project, but Tesla is doing it. Figure AI you probably know, and Apptroniks. Agility Robotics is another, and I have the highest respect for their work. There are many other companies. These are all smart business people as well. I guess there is a business case, I'm not exactly sure, but at least technology wise we're getting close. I'm not an expert in the business side, so I'll leave it there.

***Marwa ElDiwiny:* Since your vision or your dream is that we have humanoid robots in our home, do you believe that everyone should have a humanoid robot?**

**Dennis Hong:** It depends when. Today? Absolutely no. I don't trust our robots. I don't have any robots at my home. I don't trust our robots. Way in the future, without giving me a year or something like that, well, if the robot is stable and useful and economically makes sense, then yeah, why not? We all know that we build robots for what, the three Ds—the dull, dirty, dangerous—things that humans shouldn't be doing or cannot do or don't want to do, let the robots do it. Why not have a humanoid robot at home and make it do all the things that we don't want to do? The answer is yes, when we have these types of robots, which is probably still a long way to go.

***Marwa ElDiwiny:* One question about MasterChef then. So, you also have activities beyond academia and research. Do you see robots being useful in the kitchen?**

**Dennis Hong:** For people who don't know me, I'm also a hobbyist, a pretty serious gourmet chef. When friends and family come over, I do an eight-course degustation menu. I was on MasterChef USA season four as well. Don't look it up, it's kind of embarrassing, but I was on there. I cook every day at home, by the way. There are great chefs in the world. There are great roboticists in the world, but there are not many good chefs who are also roboticists. I'm one of them. I haven't really talked publicly about it, but we do have a pretty big multimillion-dollar project for a cooking robot funded by a Korean company, but we're not supposed to tell too much about it.

I can say that the project is called Project Yori, it's not a single-tasker cooking robot. Theoretically, it can cook anything. I'll just put it that way, and it doesn't cook like humans because most of the robots you've probably seen, they try to mimic how humans cook. This robot doesn't even have hands.

***Marwa ElDiwiny:* Any closing thoughts?**

**Dennis Hong:** This is for the younger generation of the general public. My interest in this field started when I was 7 years old. This is a true story. I watched the movie Star Wars, the first one, Episode IV, for the first time in the Mann's Chinese Theater, with all the lightsabers, and all the spaceships completely blew me away, but in the movie, the two droids, they call it droids, the R2-D2, the one that looks like a trash can, and C-3PO, the humanoid robot completely blew my mind. And on my way home in the car, I told my mom and dad, “I want to grow up to become a robot scientist,” and I followed my dream and I'm here today, so follow your dreams. That's what I'm doing. I'm not the richest person in the world, but I can safely say that I'm probably one of the happiest people on this planet, so follow your dreams.

***Marwa ElDiwiny:* Thank you so much. It's been a great honor talking to you, and I really appreciate it.**

